# Caregiver overload and factors associated with care provided to patients under palliative care

**DOI:** 10.17533/udea.iee.v39n1e10

**Published:** 2021-03-05

**Authors:** Alice Regina Felipe Silva, Jack Roberto Silva Fhon, Rosalina Aparecida Partezani Rodrigues, Mariane Thais Pecchi Leite

**Affiliations:** 1 Nurse. Escola de Enfermagem de Ribeirão Preto da Universidade de São Paulo, Ribeirão Preto, Brazil. Email: alice.regina.silva@usp.br Escola de Enfermagem de Ribeirão Preto da Universidade de São Paulo Ribeirão Preto Brazil alice.regina.silva@usp.br; 2 Nurse. Ph.D. Professor. Escola de Enfermagem da Universidade de São Paulo, São Paulo, Brazil. Email: betofhon@usp.br. Corresponding author. Escola de Enfermagem da Universidade de São Paulo São Paulo Brazil betofhon@usp.br; 3 Enfermeira Ph.D. Professora Titular. Escola de Enfermagem de Ribeirão Preto da Universidade de São Paulo, Ribeirão Preto, Brazil. Email: rosalina@eerp.usp.br Escola de Enfermagem de Ribeirão Preto da Universidade de São Paulo Ribeirão Preto Brazil rosalina@eerp.usp.br; 4 Nurse. Escola de Enfermagem de Ribeirão Preto da Universidade de São Paulo, Ribeirão Preto, Brazil. Email: mariane.thais.leite@usp.br Escola de Enfermagem de Ribeirão Preto da Universidade de São Paulo Ribeirão Preto Brazil mariane.thais.leite@usp.br

**Keywords:** caregivers, palliative care, home care service, cuidadores, cuidados paliativos, serviço de assistência domiciliar, cuidadores, cuidados paliativos, servicio de atención domiciliaria

## Abstract

**Objective.:**

To identify overload and associated factors among caregivers of adult patients receiving palliative care.

**Methods.:**

Descriptive, quantitative, and cross-sectional study addressing 40 adults under palliative care and their respective caregivers enrolled in the Home Care System in Ribeirão Preto, Brazil. Data concerning the patients included demographic profile and Mini-Mental State Examination. A form was used to collect the caregivers’ demographic data along with the Zarit Burden Interview Scale, Self-Reporting Questionnaire, Beck Depression Inventory, and Coping Strategies Inventory.

**Results.:**

Regarding the patients, 84.2% were women, 52.6% were over 80, 65.8% had no partner, and 76.3% presented cognitive impairment. The caregivers were mostly women (84.5%), aged 56.67 years old on average, were the patients’ children (42.5%); had no partner (55%), and lived with the patient (77.5%). The mean score obtained in the burden scale was 28.78 points, 32.5% had stress, and 42.5% depression. Regarding coping strategies, the ones most frequently used were positive reappraisal (12.8), withdrawal (10.2), and problem solution (9.7). A positive and statistically significant correlation was found between time spent with care (days and hours) and escape/avoidance with overload. Linear regression analysis revealed an association between being a woman (*p*=0.002), number of days spent with care (*p*=0.004), and depression (*p*<0.001) with overload.

**Conclusion.:**

Being a woman, spending more days providing care, and depressive symptoms were associated with caregiver overload.

## Resumen

**Objetivo.:**

Identificar la sobrecarga y los factores relacionados en el cuidador de pacientes adultos en cuidados paliativos.

**Métodos.:**

Estudio descriptivo, cuantitativo y transversal que contó con la participación de 40 adultos en cuidados paliativos atendidos en el Sistema de Atención Domiciliaria, Ribeirão Preto, Brasil y sus respectivos cuidadores principales. La recolección de la información consistió en el perfil demográfico y en el *Mini Mental State Examination*. Para el cuidador, se utilizó el perfil demográfico, la Escala de Sobrecarga de Zarit, *Self-Reporting Questionnaire*, el Inventario de Depresión de Beck y el Inventario de Estrategias de Coping.

**Resultados.:**

Con relación a los pacientes, 84.2% eran mujeres, 52.6% cuentan con una edad superior a los 80 años, 65.8% no tienen pareja y 76.3% sufren de déficit cognitivo. Los cuidadores fueron predominantemente del sexo femenino (84.5%), con una edad promedio de 56.67 años, eran hijos (42.5%), sin compañero (55%) y vivían con el paciente (77.5%). El promedio de sobrecarga fue 28.78 puntos. Además, 32.5% presentaron estrés y 42.5% depresión. Con relación al *Coping*, las estrategias más utilizadas identificadas por la media fueron reevaluación positiva (12.8), alejamiento (10.2) y resolución de problemas (9.7). Hubo correlación significativa positiva entre el tiempo de cuidado (días y horas) y la estrategia de Escape, y entre la fuga y la sobrecarga. En el análisis de regresión lineal, se identificó asociación de la sobrecarga con: sexo femenino (*p*=0.02), número de días de cuidado (*p*=0.04) y depresión (*p*<0.001).

**Conclusión.:**

Fue evidenciado que factores como ser mujer, un mayor número de días cuidando del paciente y presentar síntomas depresivos están asociados con la sobrecarga en el cuidador.

## Resumo

**Objetivo.:**

Identificar a sobrecarga e os fatores relacionados no cuidador de pacientes adultos em cuidados paliativos.

**Métodos.:**

Estudo descritivo, quantitativo e transversal com 40 adultos em cuidados paliativos e seus respectivos cuidadores principais atendidos pelo Sistema de Atenção Domiciliar, Ribeirão Preto, Brasil. A coleta das informações sobre os pacientes foram o perfil demográfico e Mini Exame do Estado Mental. Para o cuidador foi utilizado o perfil demográfico, Escala de sobrecarga de Zarit, Self-Reporting Questionnaire, Inventário de Depressão de Beck e Inventario de Estratégias de Coping.

**Resultados.:**

Quanto aos pacientes, 84.2% eram mulheres, 52.6% com idade superior de 80 anos, 65.8% sem companheiro e 76.3% com deterioro cognitivo. Os cuidadores foram predominantemente do sexo feminino (84.5%), média de idade de 56.67 anos; eram filhos (42.5%); não tinham companheiro (55%) e viviam com o paciente (77.5%). A média de sobrecarga foi de 28.78 pontos. Ademais, 32.5% apresentaram estresse e 42.5% depressão. Quanto ao Coping, as estratégias mais utilizadas identificadas pelas médias foram reavaliação positiva (12.8), afastamento (10.2) e resolução de problemas (9.7). Houve correlação significativa positiva entre o tempo do cuidado (dias e horas) e a estratégia de esquiva e fuga com a sobrecarga. Na análise de regressão linear identificou-se associação entre o sexo feminino (*p*=0.002), número de dias do cuidado (*p*=0.004) e depressão (*p*<0.001) com a sobrecarga.

**Conclusão.:**

Foi evidenciado que fatores como ser mulher, maior número de dias cuidando do paciente e apresentar sintomas depressivos estão associados com a sobrecarga do cuidador.

## Introduction

An estimated 40 million people require palliative care (PC) every year; 78% of these individuals live in developing countries. With epidemiological and demographic changes, Non-communicable Chronic Diseases (NCDs) are the leading cause of a condition in which PC is required that exposes the finitude of life while promoting autonomy during the dying process.(1) PC is defined as holistic care provided to individuals at any age, suffering from a severe illness, especially those experiencing the end of life. The objective of which is to improve the quality of life of patients and their families.(2) A patient receiving PC may present physical, psychopathological, social, or spiritual changes. These changes are even more apparent when a patient is at home, which may require changes in the family environment to accommodate care actions, emotionally affecting family members and mainly caregivers, potentially causing overload and decreased quality of life.(3) PC takes into account the patient-family pair, that is, the provider and recipient of care,(4) considered the first and most important health alliance, as this pair shares particularities and familiarity that favor the monitoring of the health-disease process.(5)

An informal caregiver is generally an individual who provides unpaid care and possibly experiences restrictions arising from the responsibility of providing care, which may lead to a condition called caregiver overload.(6) Caregiver overload is a psychological situation that results from a combination of physical strain, emotional pressure, restricted social life, and financial/economic demands determined by the process of providing care to an ill individual. Overload may become more intense when the patient is diagnosed with an incurable disease.(7) Overload may result from various factors, but it is mainly influenced by the health condition of individuals under PC. The phase causing the most intense overload is the end of life, when patients may experience pain, insufficient respiratory distress, mental confusion, anxiety, or depression, requiring caregivers to deal with these demands and bring balance into the care process.(8)

A patient receiving PC demands time from caregivers, who need to adapt their lives to the patient’s routine and needs, which may cause physical changes (back pain and loss of sleep), compromise domestic chores, lead to psychological (depression and stress) and social changes (isolation, unemployment, breaking ties) causing caregivers to experience health problems.(5) The increased number of patients requiring PC due to NCDs, or aging and increased life expectancy, has led more families to deal with the difficulties of taking care of a family member. This study is relevant because it sheds light on health demands and gives a direction to the care plan devised to the patient-family/caregiver. From this perspective, this study’s objective was to analyze caregiver overload and associated factors among the caregivers of adult or elderly patients receiving palliative care. 

## Methods

Descriptive, quantitative, and cross-sectional study conducted in the Home Care Service of the City Health Department in Ribeirão Preto, Brazil. The study’s population was recruited from the Home Care Service database, which included 150 patients, 96 of whom were receiving palliative care. Fifteen of these patients refused to participate, 12 had moved to another city, 15 had died, eight changed their phone numbers, and six were hospitalized at the time of data collection, so that 40 patients and their respective caregivers, enrolled in the Home Care Service from January to April 2019 composed the final sample. Inclusion criteria used for adult/elderly patients were: being enrolled in the Home Care Service, 18 years old or older, capable of answering the instruments or being accompanied by an informal caregiver, and receiving PC. Inclusion criteria for caregivers were: being the primary caregiver and aged 18 years old or older.

A meeting was scheduled at the patient’s home, and undergraduate and graduate students previously trained by the study’s coordinator held a 30-minute interview to collect data from both participants, using the following instruments:

### For patients

- Sociodemographic profile: information regarding the patients’ sex (male/female), age (complete years), marital status (with or without a partner), education (years of schooling), number of children, number of people living with the adult-elderly patient, and the patient’s and family’s monthly income.

- Mini-Mental State Examination (MMSE): instrument addressing cognitive function. It was translated and validated to Portuguese,(9) and its questions are grouped into seven categories. The total score ranges from zero to 30, and the cutoff points validated for the Brazilian population are: 20 points for illiterate individuals, 24 for individuals from 1 to 4 years of education, 26.5 points for individuals from 5 to 8 years of schooling, 28 for individuals from 9 to 11 years of schooling, and 29 for individuals with more than 11 years of schooling.(9)

### For caregivers

- Sociodemographic profile: addressing information such as sex (male/female), age (full years), marital status (with or without a partner), kinship, how long the caregiver has provided care to the patient, how many hours and days are spent in the care provided to the patient, and knowledge regarding the patient’s disease.

- Zarit Burden Interview Scale: translated and validated for the Brazilian culture,(10) this scale assesses perceived impact on physical and emotional health, social activities, and financial conditions. The instrument is composed of 22 questions, and its score ranges from zero to 88. There is no cutoff point; the higher the score, the greater the caregiver’s perceived overload.

- Self-Reporting Questionnaire (SRQ): developed and validated in Brazil(11), the objective of which is to detect emotional distress in the general population. It is composed of 20 close-ended questions (yes/no answers). The higher the frequency of positive answers, the more intense the emotional stress. Its score ranges from 0 to 20, with a cutoff point equal to eight(8).

- Beck Depression Inventory: developed by the American Psychiatric Association to detect depressive symptoms and later validated to Portuguese(12). It consists of 21 items composed of four statements addressing the intensity of depressive symptoms rated on a scale ranging from 0 to 3. The total score is classified as no depression (score from 0 to 10); mild to moderate depression (11 to 18), moderate to severe depression (19 to 29), and severe depression (30 to 63).

- Coping Strategies Inventory (CSI): validated to Portuguese(13) it encompasses thoughts and actions people adopt to cope with internal and external demands arising from specific stressful situations. It contains 66 questions rated on a four-point Likert scale, ranging from 0: never; 1: seldom; 2: often; 3: almost always. The items are assessed through mean scores obtained within each factor. There are eight factors: confrontation, withdrawal, self-control, social support, responsibility acceptance, escape/avoidance, problem-solving, and positive reappraisal. These factors were classified into two categories: (1) functional strategies, composed of self-control, social support, problem-solving, positive reappraisal, and responsibility acceptance, and (2) dysfunctional strategies, which correspond to confrontation, withdrawal, and escape/avoidance. Data analysis included the sum of the scores assigned to each item of the same factor, divided by the factor’s total number of items. Hence, the factors with the highest means, considered to be the most frequently used, were identified along with the items (strategies) with the highest means, that is, the strategies the study’s participants used the most.

Microsoft Excel^®^ was used to tabulate data, which were later imported to the IBM SPSS, version 25. Descriptive statistics were used along with central tendency (mean and median) and dispersion measures (standard deviation) for quantitative variables, and frequency and percentages were used for categorical variables. Additionally, the Spearman’s correlation was used to compare the means between Coping strategies and caregiver overload. The Mann-Whitney test was used to identify associations between the different factors with overload. Linear regression was used in the final analysis, with caregiver overload being the outcome variable. The significance level was established at *p*<0.05 with a 95% confidence interval for all the statistical tests.

The study was approved by the City Health Department at Ribeirão Preto and the Institutional Review Board at the University of São Paulo at Ribeirão Preto, College of Nursing (No. CAE 90111018.8.0000.5393). All the participants (patients and caregivers) signed two copies of free and informed consent forms and kept one copy.

## Results

Most of the 40 patients participating in the study were women (84.2%), aged over 80 (52.6%) with a mean of 76.5±13.8, had no partners (65.8%), lived with other family members (68.4%), and presented cognitive deficit (76.3%). The patients lived with 3.11 people on average, had 5.5 children, and a monthly income of R$1,850.63 (1 U$=R$ 4.06), while the family’s income was 3.75 times the minimum wage. Regarding the caregivers, most were women (84.5%), aged 56.7 years old on average. The caregivers were the patients’ children (42.5%), did not have a partner (55%), lived with the patient (77.5%), and had a monthly income of R$1,299.02 on average. The caregivers had provided care for an average of 82.66 months and spent 6.73 days and 20.62 hours/day providing care to patients.

Regarding caregiver overload, a mean of 28.78 points was found. Additionally, 32.5% of the caregivers experienced stress, and 42.5% presented some depressive symptoms. Regarding coping strategies, the caregivers most frequently used positive reappraisal (mean=12.88), withdrawal (10.25), and problem-solving (9.78) (Table 1).

**Table 1:** Caregiver assessment according to the Zarit Burden Interview Scale, Self-Reporting Questionnaire, Beck Depression Scale, and Coping Strategies Inventory

Variable	Descriptive statistics
Overload; mean±SD	28.8 ± 19.7
Self-Reporting Questionnaire; number (%)	
No stress	27 (67.5)
With stress	13 (32.5)
Beck Scale; number (%)	
No Depression	23 (57.5)
Mild to moderate Depression	6 (15)
Moderate to severe Depression	6 (15)
Severe Depression	5 (12.5)
Coping Strategies; Mean±SD	
Positive reappraisal	12.9±6.3
Withdrawal	10.3±4,6
Problem-solving	9.78±5.2
Self-control; Mean±SD	9.77±4.4
Social support	8.15±4.6
Confrontation	6.58±3.8
Escape and avoidance	6.48±4.6
Acceptance responsability	4.97±3.3

Analysis of the correlation between the patients’ and caregivers’ variables, relationship with care, and coping strategies with overload scale, revealed that the time spent providing care and the number of days and hours providing care presented a low positive correlation. The escape/avoidance strategy presented a statistically significant moderate positive correlation (Table 2).

**Table 2:** Correlation between the patients’ variables, caregivers’ variables, and coping strategies with caregiver overload

Variable	Correlation	***p*-value**
Patient’s age	-0.007	0.96
Caregiver’s age	0.192	0.23
Caregiver’s schooling	-0.068	0.67
How long caregiver provides care	0.310	0.05
How many days/week	0.343	0.03
How many hours/day	0.318	0.04
Coping strategies		
Confrontation	0.087	0.59
Withdrawal	0.201	0.21
Self-control	-0.110	0.50
Social support	-0.201	0.21
Responsibility acceptance	0.102	0.53
Escape and avoidance	0.421	<0.001
Problem-solving	-0.070	0.66
Positive reappraisal	-0.067	0.68

Some variables were associated with the score obtained in caregiver overload, such as the patient’s marital status (patients without a partner lead to more significant overload), caregiver’s sex (women experience more overload than men), and caregivers experiencing stress or depressive symptoms (Table 3).

**Table 3:** Comparison of the means between the patients’ and caregivers’ variables, Self-Reporting Questionnaire, and Depression with overload among caregivers of adult and elderly patients receiving palliative care

Variable	Category	Mean	***p-*value**
Patient’s sex	Male	21.6	0.60
	Female	19.6	
Patient’s marital status	Without partner	15.5	0.04
	With partner	23.1	
Cognitive status	Without deficit	25.3	0.13
	With deficit	18.9	
Caregiver’s sex	Male	11.0	0.01
	Female	22.5	
Caregiver’s marital status	Without partner	19.8	0.74
	With partner	21.0	
Self-Reporting Questionnaire	Without stress	16.5	<0.001
	With stress	28.6	
Depression	Without depression	16.5	0.01
	With depression	25.8	

The model’s goodness of fit revealed that the variables being a woman, number of days providing care to patients, and depressive symptoms are factors related with caregiver overload (Table 4).

**Table 4:** Association between the study’s variables and caregiver overload

Variables	β	CI 95% do (	***p*-value**
Constant	-60.81	-117.89 - -3.73	0.03
Female caregiver (vs. male caregiver)	15.56	2.27 - 28.84	0.02
Number of days providing care	7.47	1.36 - 14.58	0.04
Total Beck depression inventory	1.91	1.43 - 1.99	<0.001

## Discussion

The progression of the illness of a patient under PC, even with long-term treatment for chronic disease, requires health care for life. Thus, the family of a patient receiving PC needs to adapt its daily routine to meet this patient's health needs, especially the primary caregivers who provide care for most of the time. This study shows that most caregivers were women, had children, had no partner, lived with the elderly individual, and obtained a mean overload score of 28.78 points. Additionally, caregiver overload was associated with being a female caregiver, the number of days providing care, and depressive symptoms.

The demographic profile of caregivers is similar to that presented by other studies addressing this topic,(14^,^^15^) in which results are related to the caregivers’ sex and kinship to the patient, though different studies found that caregivers had a partner/spouse.(3^,^^15^) In a still sexist society, families experiencing the need to provide care usually assign this responsibility to daughters, who, in addition to meet the needs of a patient, also assume other responsibilities within the family. Even though daughters assume the responsibility to provide care, many caregivers are older women taking care of other elderly individuals.(16) Nonetheless, being the patient’s child, caregivers play an essential role within the family, and in most cases, need to assume this responsibility as they are the only child, single, and have no one else to share this duty.(17)

The mean score obtained in the Zarit Burden Interview Scale(14) was 28.78 points. Brazilian authors verified a lower mean (17.88) among caregivers located in São Paulo, Brazil.(14) Another study conducted in the Home Care Service located in Porto Alegre, Brazil, addressing 80 caregivers of adult patients, verified a mean overload score of 41.04 points.(18)

Caregiver overload cause changes in the relationship established with the family, at work, income, leisure, and in the caregiver’s mental and physical health.(3) This overload is linked to the patient’s illness.(15) Primary caregivers are seldom prepared to assume all the responsibilities that are placed on them and often have to face unexpected situations and tasks that require health workers to provide proper guidance.(19)

Overload was correlated with the escape/avoidance strategy. In one study conducted with 225 caregivers, the authors identified that the three most frequently used strategies were self-control, positive reappraisal, and planned problem-solving, which alleviated caregiver overload and improved the care plan of patients undergoing hemodialysis, while confrontation and escape/avoidance were the least used strategies.(20) The escape/avoidance strategy is related with an attempt of caregivers to deny the current situation of their family member, not being able to overcome the challenging situation, and experiencing negative feelings, especially regarding the patient’s death.(21)

Data analysis showed an association between caregiver stress and overload. The long-term disease of a patient receiving care at home is a situation that leads to stress, threatening an individual’s personal, familiar, and social balance. Lack of balance leads caregivers to experience problems, as they no longer have a problem-solving mechanism, experiencing disorganization and negative feelings such as fear, guilt, and anxiety.(22)

Women also experienced more intense overload compared to male caregivers. The hypothesis is that family care dynamics changed after women entered the job market. In addition to working outside the home, women assume the role of mothers, wives, and homemakers; that is, women assume an excess of responsibilities, which, combined with the caregiver role, can lead to overload.(23)

An association was found between the number of days providing care and caregiver overload. Delalibera *et al.*(14) report that caregivers provided care for an average of 24 months, and 38.3% presented moderate overload. In this context, in which caregivers spend many hours providing care to patients and often relegate the care of their home, self-care is compromised, and their health may be harmed.(5^,^^8^) Additionally, when caregivers cannot perform daily tasks due to a lack of time, they break family bonds and spend less time socializing with friends, at work, or enjoying leisure time. This change in routine, which is adapted to provide care to someone else, generates frustration and potential physical and emotional overload, that is, caregivers abdicate their own needs and interests to provide care, even though no psychological or material support is provided, which in turn, may lead to depression and stress.(24)

The relationship between depression and caregiver overload may be influenced by various sociocultural factors such as sex, age, race, lack of social support, which may influence how caregivers respond to overload,(25) harming the family’s functioning. Hence, this study identified that depressive symptoms are associated with caregiver overload.

This study has two limitations: 1. The participants were recruited from a database provided by a Home Care Service, which presented inconsistent data, hindering the identification and retrieval of information; 2. The sample's small size may be related to the population's particular characteristics so that inferences concerning this study's results cannot be generalized to other populations. Data analysis revealed that the factors associated with caregiver overload were being a woman, number of days providing care to patients, and depressive symptoms.

This study contributes to scientific knowledge concerning overload among the caregivers of patients receiving palliative care and monitored by a Home Care Service at home. Attention should be paid to the cultural, historical context in which this responsibility is assigned to female caregivers, to the fact that the mental health of caregivers is often neglected, and on how nurses have aided these caregivers to manage their health. Therefore, future studies are recommended to address a larger sample of caregivers of patients under palliative care and implement follow-up to devise strategies, care plans, and interventions intended to decrease caregiver overload and later become part of palliative care protocols. It is crucial that nursing workers are attentive to the health needs of patients and caregivers, heeding the needs of caregivers and enabling them to provide quality and effective care to patients receiving PC.
